# Perampanel as first add-on antiseizure medication: Italian consensus clinical practice statements

**DOI:** 10.1186/s12883-021-02450-y

**Published:** 2021-10-26

**Authors:** Paolo Bonanni, Antonio Gambardella, Paolo Tinuper, Benedetto Acone, Emilio Perucca, Giangennaro Coppola

**Affiliations:** 1 IRCCS Eugenio Medea Scientific Institute, Epilepsy Unit, Conegliano, Via Costa Alta 37, 31015 Conegliano, TV Italy; 2 Institute of Neurology, University Magna Graecia, Catanzaro, Italy; 3 IRCCS Istituto delle Scienze Neurologiche di Bologna, Bologna, Italy; 4 Department of Biomedical and Neuromotor Sciences, University of Bologna, Bologna, Italy; 5 Cartesio solutions s.r.l, Venezia, Italy; 6 Division of Clinical and Experimental Pharmacology, Department of Internal Medicine and Therapeutics, University of Pavia, Pavia, Italy; 7 Department of Neuroscience, Monash University, Melbourne, Australia; 8 Department of Medicine, Surgery, Odontoiatry, Medical School of Salerno, University of Salerno, Salerno, Italy

**Keywords:** Perampanel, Epilepsy, Delphi procedure, Antiepileptic drugs, Adjunctive therapy

## Abstract

**Background:**

When use of a single antiseizure medication (ASM) fails to induce seizure remission, add-on therapy is justified. Perampanel (PER) is approved in Europe as adjunctive therapy for focal, focal to bilateral tonic-clonic seizures and generalized tonic-clonic seizures. Aim of the study was to establish whether PER is suitable for first add-on use.

**Methods:**

A Delphi methodology was adopted to assess consensus on a list of 39 statements produced by an Expert Board of 5 epileptologists. Using an iterative process, statements were finalized by a Delphi Panel of 84 Italian pediatric and adult neurologists. Each statement was rated anonymously to determine level of agreement on a 9-point Likert scale. Consensus was established as agreement by at least 80% of the panelists. The relevance of each statement was also assessed on a 3-point scale.

**Results:**

Consensus was achieved for 37 statements. Characteristics of PER considered to justify its use as first add-on include evidence of a positive impact on quality of life based on long term retention data, efficacy, tolerability, and ease of use; no worsening of cognitive functions and sleep quality; a low potential for drug interactions; a unique mechanism of action. Potential unfavorable factors are the need for a relatively slow dose titration; the potential occurrence of behavioral adverse effects; lack of information on safety when used in pregnancy; limited access to plasma PER levels.

**Conclusion:**

Perampanel has many features which justify its use as a first add-on. Choice of an ASM as first add-on should be tailored to individual characteristics.

**Supplementary Information:**

The online version contains supplementary material available at 10.1186/s12883-021-02450-y.

## Background

In about 50% of patients, the antiseizure medication (ASM) used initially fails to induce sustained seizure remission [1]. In these patients, management options involve use of an alternative monotherapy or a combination therapy. There is no evidence that either strategy is superior to the other [2, 3].

When a first add-on strategy is preferred, the selection of the ASM is not simple. When all factors that need to be taken into account are considered [4], it is clear that none of the available ASMs is ideal for use as a first add-on in all patients. Yet, it is important for physicians to be aware of the characteristics of each ASM, and of the criteria to be used in deciding to what extent such characteristics should favor or discourage first add-on use in individual cases.

In this article, we used a Delphi approach to produce a consensus document outlining to what extent perampanel (PER) meets the characteristics that would favor its first add-on use. Perampanel is approved in Europe as adjunctive therapy of focal and focal to bilateral tonic-clonic seizures (FBTCS) in patients aged ≥4 years, and as adjunctive treatment of generalized tonic-clonic seizures (GTCS) in patients aged ≥7 years with idiopathic generalized epilepsy (IGE).

## Methods

The Delphi process is a well-established methodological tool which is used to assess and integrate the opinions of experts in areas where univocal evidence from well design studies is unavailable [5]. The procedure used to produce the present document has been described in detail in a previous publication [4]. In short, an Expert Board of 5 epileptologists produced initially a list of 39 statements relevant to the aims of the work. Using an iterative process, statements were finalized by a Delphi Panel of 84 Italian pediatric and adult neurologists (Appendix). Each statement was rated anonymously to determine level of agreement on a 9-point Likert scale. Consensus was established as agreement by at least 80% of panelists. The relevance of each statement was also assessed on a 3-point scale.

## Results

Seventy-four of the 84 members of the Delphi Panel (87%) completed all rounds of the rating procedures. Thirty six of the 39 initial statements reached consensus in the first round. The remaining three statements were modified based on feedback received in the first round, and, of these, one achieved consensus later in the process. Therefore, consensus was ultimately reached for 37 (95%) of the 39 statements. Clinical relevance received a mean rating above 2 (on 3-point scale) for all of the statements (Supplementary Tables 1–3).

### General statements

*Overall, the Panel agreed that PER has a positive impact on quality of life based on evidence of favorable short- and long-term retention data in clinical studies, efficacy, tolerability, safety and ease of use (Supplementary Table* *1**). Quality of life, efficacy, tolerability, safety and ease of use are primary considerations when selecting an ASM as first add-on* [4, 6–8]*.*

The above statement is supported by results obtained in randomized double-blind trials [9–14] and observational studies [15, 16]. In particular, in an open-label extension study that followed up patients with focal seizures, retention rates were found to be 46% at 3 years and 39% at 4 years [11], which are similar to those reported in studies that evaluated retention for other ASMs for 2 or more years [17, 18].

Statements related to specific areas are discussed below.

### Statements related to efficacy

*There was consensus among Panel members that PER is effective against focal and GTCS and does not worsen other types of seizures, such as absence or myoclonic seizures.*

In randomized double-blind trials, responder rates recorded in adolescents and adults have been found to be significantly greater with PER than with placebo for focal seizures, FBTCS, and GTCS [9, 10, 12]. More recently, Brandt et al. [19] in a post hoc analysis found that PER does not exacerbate absence seizures or myoclonic jerks in patients with IGE. In particular, the number of patients who developed “de novo” myoclonic and/or absence seizures during PER treatment was the same as in the placebo-treated patients.

Of note, the efficacy and safety profile of PER reported during long-term follow-up studies is similar to that observed in double-blind, phase III studies [11, 13]. A recent global, multicenter, open-label, single-arm study provided evidence for PER being also safe and effective in the adjunctive therapy of focal seizures, focal to FBTCS and GTCS in children age 4 to < 12 years [14].

*There was consensus that* PER *may be useful when there is uncertainty on whether seizures are focal or generalized.*

Because PER is effective against focal as well as GTCS, its use may be considered for patients who experienced one or more tonic-clonic seizures that could not be readily classified as being of either focal or generalized onset [20]. Such diagnostic uncertainty is especially common in childhood epilepsies [21]. The feasibility of using a broad-spectrum ASM in this situation, however, should not lead to neglect further investigations to diagnose the correct seizure type and syndrome.

*There was consensus that PER can be effective and well tolerated when used either early or late as add-on therapy, even at low doses.*

The results of a post-hoc analysis of Phase III trials indicate that adjunctive treatment with PER is associated with greater efficacy in patients who are taking fewer concomitant ASMs [22]. A pooled post hoc analysis of four randomized studies [23] also showed that adjunctive therapy with PER can be efficacious at low doses (4 mg/day). Of note, the improvement in seizure control at a dose of 4 mg/day was greater in patients not receiving enzyme inducing ASMs, whereas the occurrence of adverse effects (AEs) at that dose was similar in patients on and off enzyme inducers [23]. Recent multicenter open-label prospective trials reported that use of PER as first add-on at a median dose of 6 mg/day was associated with improved control of focal seizures, with or without evolution to FBTCS, as well as GTCS [15, 16].

### Statements related to safety and tolerability

*There was consensus that PER has a favorable short- and long-term safety and tolerability profile. There was also consensus that, like other ASMs, PER can cause dose-related AEs, the most frequent of which are dizziness, somnolence, headache, fatigue, and irritability, and that these dose-related AEs, especially those affecting the central nervous system (CNS), occur more commonly in the first weeks and tend to decrease over time with the continuation of therapy. The Panel also agreed that PER tolerability is improved when fewer concomitant ASMs are used, and when PER is administered at low doses and with a slow titration.*

Post hoc analyses of data from pooled Phase III studies have shown that most AEs of PER occur during the titration phase and tend to subside within a few weeks [24]. A post-hoc analysis of data from Phase III studies has also suggested that tolerability is improved when PER is used as an early add-on treatment [22]. More recent prospective or retrospective studies have reported that, when PER is prescribed as first add-on, the frequency of AEs is halved, especially when low doses (≤ 6 mg) and a slow titration (2 mg/day every > 2 weeks) are used [15, 16].

*There was consensus that a decision to use PER should take into consideration any comorbidities present at baseline, as for other ASMs. In consideration of PER’s behavioral and psychiatric tolerability profile (mainly irritability), there was consensus about the need to carefully evaluate whether PER is indicated in patients who present with these problems at baseline or in their medical history, and to monitor clinical response should the drug be prescribed in these patients. There was also consensus that when PER is used as first add-on therapy, it may be possible to reduce the dose of concomitant ASMs in order to optimize tolerability.*

The retrospective Fydata study has shown that patients with prior psychiatric comorbidities are more likely to experience psychiatric AEs with PER [25]. However, Hasegawa and Tohyama [26] in another retrospective study reported that PER may either aggravate or ameliorate psychiatric and behavioral symptoms, and that, in particular, improvement may occur in seizure-free patients. In a cohort of patients with drug-resistant focal epilepsy, PER did not increase the baseline level of irritability, depression, or anxiety [27]. Additionally, many real-life retrospective and prospective studies have indicated that use of slow titration schedules (2 mg/3–4 weeks) and low doses are associated with a lower risk of psychiatric AEs, especially when PER is used as first add-on [15, 16, 25].

*The Panel agreed that, in either adolescents or adults, significant AEs of PER on cognitive functions have not been demonstrated in the short- and medium-term.*

The evidence supporting this statement comes from a double blind, randomised study [28] and 4 observational prospective studies [14, 29–31]. ASM-induced cognitive AEs are one of the main detrimental factors for the quality of life of people with epilepsy. The favorable cognitive profile of PER is, therefore, important in this regard.

*There was consensus that, although sleepiness is an* AE *associated with the use of PER, assessment of sleep through specific tests has shown that PER does not worsen daytime sleepiness and the quality of night sleep in most patients.*

The data published by Rocamora et al. [32] suggest that PER can improve the quality of sleep by having a favorable effect on several sleep parameters, without worsening daytime sleepiness. Sleep disorders in patients with epilepsy have a high prevalence and poor sleep quality or duration can worsen seizure control and, consequently, quality of life [32].

*There was consensus that PER does not affect adversely cardiac electrophysiology.*

A Phase I thorough QT study and a pooled analysis of 3 Phase III studies have shown no evidence of prolonged QT interval duration with PER treatment [33].

*There was consensus that there are currently no data on the safety of PER in pregnancy, particularly with regard to seizure control and possible AEs on the offspring.*

Following the completion of the Delphi Panel, a report of 96 pregnancies exposed to PER has been published [34]. However, the data presented are insufficient to make any meaningful estimate of potential risks for the mother or the offspring.

### Concomitant ASMs and drug interactions

*Perampanel is indicated for add-on therapy. PER is metabolised in the liver and is subject to enzyme induction. There was consensus that, when adding PER to a pre-existing monotherapy, it is worth considering whether or not an inducer is present because a higher dose of PER may be needed.*

A number of enzyme inducing ASMs (carbamazepine, oxcarbazepine and phenytoin) have been found to stimulate PER metabolism and to reduce the plasma exposure to PER without affecting the relationship between plasma PER concentration and clinical response [35, 36]. In a post hoc analysis of randomized trials, seizure reduction at PER doses of 8 and 12 mg/day was significantly greater in patients receiving non-enzyme inducing ASMs than in patients receiving enzyme inducers [37]. The incidence of some AEs, however, was also greater in patients on non-enzyme inducing ASMs. For patients taking enzyme inducing ASMs, any decrease in efficacy due to reduced exposure could be compensated for by increasing the dose of PER [37, 38]. According to European and U.S. prescribing information, however, a dose of 12 mg/day should not be exceeded even in the presence of enzyme inducers, because there is insufficient information on the use of higher doses [39, 40].

*The Panel agreed that there is no evidence that the risk of AEs is increased when PER is combined with specific ASMs or ASM classes. There was also consensus that to optimize PER tolerability it may be possible to reduce the dosage of concomitant ASMs.*

In some real life studies, the introduction of PER has permitted to simplify the ASM regimen and, in some cases, to convert patients to PER monotherapy [15, 41].

*The Panel agreed that PER has a limited number of drug interactions, generally of modest clinical significance. No drug interactions leading to a worsening of the tolerability of PER have been reported to date.*

As discussed above, concomitantly administered carbamazepine, phenytoin and oxcarbazepine decrease plasma PER concentrations to an extent that can be clinically significant. Moreover, PER has been found to cause a modest (< 10%) decrease in the plasma concentration of carbamazepine, clobazam, lamotrigine, and valproic acid [39], but these interactions are not expected to be of clinical significance.

At a dose of 12 mg/day, but not 4 or 8 mg/day, PER decreases by about 40% the plasma concentration of levonorgestrel, potentially resulting in contraceptive failure [39].

### Mechanism of action

*There was consensus that PER has a unique mechanism of action, which is complementary to that of other ASMs currently on the market.*

Perampanel is a noncompetitive selective antagonist of the α-amino-3-hydroxy-5-methyl-4-isoxazolepropionic acid (AMPA) receptor. AMPA receptors mediate postsynaptic responses to glutamate, the primary excitatory neurotransmitter in the CNS, and are thought to play a crucial role in the generation and spread of epileptic activity [42].

When selecting an ASM for adjunctive therapy, the mechanism of action is a potentially important consideration, especially when adding a first add-on drug [4]. Evidence from preclinical and clinical studies suggests that combinations of ASMs with different mechanisms lead to improved outcomes compared with combinations of ASMs acting by the same mechanism [43–46].

### Statements related to adherence issues

*There was consensus that PER shows several properties facilitating a good adherence,* i.e. *a long plasma half-life that allows once daily administration, a simple dosing schedule (one tablet for each dosing level), and the convenience of single dosing at bedtime. The statement that regular compilation of the therapeutic plan can improve therapeutic adherence failed to reach the threshold for consensus among Panel members.*

Over one-third of patients with epilepsy are non-adherent to the prescribed treatment regimen, and suboptimal adherence is an important cause of persisting seizures [47], as well as a risk factor for emergency department visits, hospital admissions, injuries, and even mortality [48], possibly due to SUDEP [49]. ASMs that need to be taken less frequently have been consistently associated with better adherence [50]. The therapeutic plan is a requirement set by Italian Health Authorities for certain medicines. It requires prescriptions to be registered in a file which, in the case of PER, must include the patient’s personal data and the eligibility criteria for the correct prescription of the medication as stated by the summary of product characteristics. However, it is not a specific purpose of the therapeutic plan to improve adherence to therapy.

### Ease of use of the medication and monitoring procedures

*There was consensus that PER is associated with several factors associated with ease of use, such as a simple one-tablet once daily dosing regimen, availability of different oral formulations, effectiveness at low doses in some patients, and no requirement for repeated routine blood chemistry or laboratory investigations in most patients. The Panel also agreed that, should therapeutic drug monitoring be desirable, measurements of plasma PER levels are not widely accessible.*

Overall, PER has several characteristics considered to be desirable for a first add-on use ASM, including a simple once-daily dosing regimen, the availability of both liquid and solid oral dosage forms, and no need for intrusive monitoring [4]. There are, however, other desirable properties that are not met by PER, such as the need for relatively slow titration, the lack of parenteral formulations, and the lack of wide access to services for measuring plasma PER levels, which could aid in assessing adherence [4].

### Peculiarities to the Italian setting

*The Panel agreed that distribution through the DPC channel (*i.e. *the acquisition and distribution of medicines to pharmacies handled by local health authorities) guarantees PER availability and avoids medication shortage problems. The Panel also agreed that the pricing system for PER in Italy guarantees the same cost to the national health service irrespective of the prescribed daily dose. There was also consensus that the distribution of drugs such as PER, that are included in the PHT formulary (hospital-district level formulary), guarantees greater cost-effectiveness for the health service compared to non-PHT drugs, which mainly use the traditional distribution channel. However, there was no consensus that compilation of the therapeutic plan can improve the appropriateness of PER prescribing.*

The statements listed above reflect measures that are in place to facilitate access to, and a more cost-effective use of medications, such as PER, which are distributed via the PER-PHT channel. The therapeutic plan is a requirement set by Italian Health Authorities. Although one purpose of the therapeutic plan is to facilitate the appropriate use of medicines, the statement that prescribing appropriateness is improved by the therapeutic plan just failed to reach the threshold for consensus (Supplementary Table 3).

## Discussion

In a previous document, we defined the ideal pharmacological and clinical characteristics which favor utilization of an ASM as first add-on in patients with epilepsy unresponsive to monotherapy [4]. We have now applied a Delphi procedure to finalize a consensus document to determine to what extent PER meets such characteristics.

The Panel agreed that the efficacy of PER as adjunctive treatment of focal seizures and GTCS has been clearly demonstrated, and that there is no evidence for PER causing a worsening of other seizure types such as absence or myoclonic seizures. Because of this, PER was considered to provide an option for the adjunctive treatment of patients in whom there is uncertainty on whether seizure onset is focal or generalized. There was also consensus that PER can be effective at low doses and is generally well tolerated when used either early or late in the treatment algorithm. Most importantly, there was consensus that PER’s favorable short- and long-term retention demonstrated in clinical trials and observational studies, together with its efficacy, tolerability, safety and ease of use, are indicative of a positive impact on quality of life, and are consistent with the use of PER as first add-on medication. Additional desirable characteristics of PER which are relevant to first add-on use include a simple, one-tablet once daily dosing scheme facilitating adherence; availability of solid and liquid oral formulations; evidence for a positive effect on the quality of night sleep; evidence of lack of negative impact on cognitive functions; and no need for intrusive safety monitoring procedures. On the other hand, properties which are not ideal for first add-on use include the need for relatively slow dose titration; the potential occurrence of behavioral and psychiatric AEs, particularly in patients with a history of such problems; lack of information on maternal and fetal safety when used in pregnancy; and limited availability of services for measuring plasma PER levels.

There was also agreement that PER shows a better tolerability when it is used in combination with fewer ASMs, at low doses such as 4 and 6 mg /day and with a slow titration. A desirable feature of PER which is relevant to add-on use is its unique mechanism, which facilitates use in combination with any ASM. PER also has a low interaction potential, although its susceptibility to enzyme induction and the possible need for higher doses in patients comedicated with certain enzyme inducing ASMs was acknowledged.

## Conclusions

Based on the points highlighted in the present consensus paper, it can be concluded that PER shows many characteristics favoring first add-on use in patients with focal or GTCS not adequately controlled on monotherapy. While PER may represent a valuable option for such patients, it should be emphasized that ultimately the choice of the ASM to be used preferentially as first add-on should be tailored to individual characteristics.

### Supplementary Information

**Additional file 1: Supplementary Table 1**. General statements and statements addressing efficacy and safety issues, with associated ratings for relevance and level of agreement. **Supplementary Table 2**. List of statements addressing potential implications of drug interactions, mechanism of action and adherence, with associated ratings for relevance and level of agreement. **Supplementary Table 3**. List of statements addressing potential implications of ease of use and other factors specific for the Italian setting, with associated ratings for relevance and level of agreement.

## Data Availability

Not applicable.

## Appendix

### List of Delphi Panel members

Umberto Oscar Aguglia (Ospedali Riuniti, Reggio Calabria), Francesca Anzellotti (Ospedale SS Annunziata, Chieti), Carla Arbassino (Ospedale Salesi, Ancona), Daniela Audenino (Ospedale Galliera, Genova), Valeria Badioni (UO Università di Milano), Irene Bagnasco (Ospedale Martini, Torino), Emanuele Bartolini (Nuovo Ospedale S. Stefano, Prato), Yerma Bartolini (Ospedale Bufalini, Cesena), Vincenzo Belcastro (Ospedale S. Anna, Como), Patrizia Bergonzini (Policlinico NPI, Modena), Pia Bernardo (Univ. Della Campania Luigi Vanvitelli, Napoli), Giovanni Boero (Ospedale Centrale SS Annunziata, Taranto), Alice Bonuccelli (Ospedale Santa Chiara, Pisa), Francesco brigo (Ospedale Tappeiner, Merano), Gaetano Cantalupo (Ospedale Maggiore Borgo Trento, Verona), Giuseppe Ceres (ASL, Salerno), Caterina Cerminara (Policlinico Tor Vergata, Roma), Valentina Chiesa (Ospedale San Paolo, Milano), Antonietta Coppola (Università Federico II, Napoli), Duccio Maria Cordelli (Policlinico Sant’Orsola, Bologna), Autilia Cozzolino (Ospedale Giuseppe Moscati, Avellino), Barbara Cruciatti (Ospedale Civile, Pordenone), Valentina De Giorgis (Istituto Neurologico Mondino, Pavia), Giovanni De Maria (Spedali Civili, Brescia), Fernando De Paolis (Ospedale Santa Caterina Novella, Galatina), Roberto De Simone (Policlinico Sant’Eugenio, Roma), Francesco Deleo, (IRCCS Istituto Besta, Milano), Giuseppe D’Orsi (Ospedali Riuniti, Foggia), Vania Durante (pres. Brindisi Di Summa-Perrino, Brindisi), Raffaella Fagioli (Ospedale, Cona, Ferrara), Elisa Fallica (Ospedale, Cona, Ferrara), Elena Freri (IRCCS Istituto Besta, Milano), Alfonso Giordano (Univ. della Campania Luigi Vanvitelli, Napoli), Loretta Giuliano (Pres. Ospedaliero Gaspare Rodolico, Catania), Shalom Haggiag (Ospedale San Camillo, Roma), Francesca Izzi (AOU Tor Vergata, Roma), Angela La Neve (Ospedale Consorziale Policlinico, Bari), Emilio Le Piane (Ospedale Pugliese, Catanzaro), Concetta Luisi (Policlinico Ospedaliero, Padova), Greta Marcorig (Ospedale Civile, Gorizia), Carlo Alberto Mariani (ASP, Palermo), Carla Marini (Ospedale Salesi, Ancona), Alfonso Marrelli (Osp. Reg, S, Salvatore, L’Aquila), Marta Maschio (Policlinico Univ. Campus Bio-Medico, Roma), Roberto Michelucci (Ospedale Bellaria, Bologna), Fabio Minicucci (IRCCS San Raffaele, Milano), Antonio Modica (ARNAS Civico Di Cristina, Palermo), Maurizio Montalto (AO Riuniti Villa Sofia Cervello, Palermo), Francesca Muzzi (Policlinico Grassi, Ostia, Roma), Rosaria Nardello (Policlinico, Univ. Giaccone, Palermo), Alessandro Orsini (Ospedale Santa Chiara, Pisa), Nicola Paciello (Ospedale San Carlo, Potenza), Mariangela Panebianco (Opsedale Garibaldi, Catania), Irene Pappalardo (Ospedale San Martino, Genova), Daniela Passerelli (Ospedale, Faenza, Ravenna), Giada Pauletto (Ospedale S.M. Misericordia, Udine), Piero Penza (AOU Ruggi D’Aragona, Salerno), Gabriella Perri (Ospedale Salvini Rhodenze, Garbagnate, Milano), Marianna Pezzella (Ospedale Cardarelli, Napoli), Piero Pignatta (Ospedale Gradenigo, Torino), Dario Pruna (AO Brotzu, Cagliari), Stefano Quadri (Ospedale Giovanni XXIII, Bergamo), Rosaria Renna (AORN Cardarelli, Napoli), Sara Renzi (Ospedale Madonna del Soccorso, Ascoli), Paolo Ricciardelli (Ospedale, Faenza, Ravenna), Romana Rizzi (AO Santa Maria Nuova, Reggio Emilia), Angelo Russo (Ospedale Bellaria NPI, Bologna), Nicola Sciscio (Centro Australia, Avellino), Vittorio Sciurricchio (Policlinico San Paolo, Bari), Carlotta Spagnoli (AO Santa Caterina Nuova, Reggio Emilia), Orazio Spitaleri (Pres. Osp. *S. Marta* S. Venera, Acireale, Catania), Pasquale Striano (Istituto Gaslini, Genova), Elena Tartara (Istituto Neurologico Mondino, Pavia), Lidia Urso (AO Sant’Antonio Abate, Erice, Trapani), Anna Vaudano (Ospedale, Baggiovara, Modena), Pier Angelo Veggiotti (Ospedale Buzzi, Milano), Fabiana Vercellino (Ospedale San Biagio, Alessandria), Maurizio Viri (Ospedale Maggiore, Novara), Roberta Vittorini (OIRM Regina Margherita, Torino), Cristina Zammarchi (Ospedale Infermi, Rimini), Clara Zanchi (Ospedale, Seriate, Bergamo), Tiziano Zanoni (Osp. Borgo Trento, Verona), Luciana Zinno (Ospedale Maggiore, Parma), Leila Zummo (ARNAS Civico Di Caterina, Palermo).
