# Correction: Perampanel as first add-on antiseizure medication: Italian consensus clinical practice statements

**DOI:** 10.1186/s12883-022-02701-6

**Published:** 2022-05-17

**Authors:** Paolo Bonanni, Antonio Gambardella, Paolo Tinuper, Benedetto Acone, Emilio Perucca, Giangennaro Coppola

**Affiliations:** 1 IRCCS Eugenio Medea Scientific Institute, Epilepsy Unit, Conegliano, Via Costa Alta 37, 31015 Conegliano, TV Italy; 2 Institute of Neurology, University Magna Graecia, Catanzaro, Italy; 3 IRCCS Istituto delle Scienze Neurologiche di Bologna, Bologna, Italy; 4 Department of Biomedical and Neuromotor Sciences, University of Bologna, Bologna, Italy; 5 Cartesio solutions s.r.l, Venezia, Italy; 6 Division of Clinical and Experimental Pharmacology, Department of Internal Medicine and Therapeutics, University of Pavia, Pavia, Italy; 7 Department of Neuroscience, Monash University, Melbourne, Australia; 8 Department of Medicine, Surgery, Odontoiatry, Medical School of Salerno, University of Salerno, Salerno, Italy

**Correction: BMC Neurol 21, 410 (2021)**

**https://doi.org/10.1186/s12883-021-02450-y**

Following publication of the original article [1], an error was identified in the Appendix section, List of Delphi Panel members.

The updated appendix is given below and the changes have been highlighted in bold.

**Appendix**

**List of Delphi Panel members**

Umberto Oscar Aguglia (Ospedali Riuniti, Reggio Calabria), Francesca Anzellotti (Ospedale SS Annunziata, Chieti), Carla Arbassino (Ospedale Salesi, Ancona), Daniela Audenino (Ospedale Galliera, Genova), Valeria Badioni (UO Universita di Milano), Irene Bagnasco (Ospedale Martini, Torino), Emanuele Bartolini (Nuovo Ospedale S. Stefano, Prato), Yerma Bartolini (Ospedale Bufalini, Cesena), Vincenzo Belcastro (Ospedale S. Anna, Como), Patrizia Bergonzini (Policlinico NPI, Modena), Pia Bernardo (Univ. Della Campania Luigi Vanvitelli, Napoli), Giovanni Boero (Ospedale Centrale SS Annunziata, Taranto), Alice Bonuccelli (Ospedale Santa Chiara, Pisa), Francesco brigo (Ospedale Tappeiner, Merano), Gaetano Cantalupo (Ospedale Maggiore Borgo Trento, Verona), Giuseppe Ceres (ASL, Salerno), Caterina Cerminara (Policlinico Tor Vergata, Roma), Valentina Chiesa (Ospedale San Paolo, Milano), Antonietta Coppola (Universita Federico II, Napoli), Duccio Maria Cordelli (Policlinico Sant’Orsola, Bologna), Autilia Cozzolino (Ospedale Giuseppe Moscati, Avellino), Barbara Cruciatti (Ospedale Civile, Pordenone), Valentina De Giorgis (Istituto Neurologico Mondino, Pavia), Giovanni De Maria (Spedali Civili, Brescia), Fernando De Paolis (Ospedale Santa Caterina Novella, Galatina), Roberto De Simone (Policlinico Sant’Eugenio, Roma), Francesco Deleo, (IRCCS Istituto Besta, Milano), Giuseppe D’Orsi (Ospedali Riuniti, Foggia), Vania Durante (pres. Brindisi Di Summa-Perrino, Brindisi), Raffaella Fagioli (Ospedale, Cona, Ferrara), Elisa Fallica (Ospedale, Cona, Ferrara), Elena Freri (IRCCS Istituto Besta, Milano), Alfonso Giordano (Univ. della Campania Luigi Vanvitelli, Napoli), Loretta Giuliano (Pres. Ospedaliero Gaspare Rodolico, Catania), Shalom Haggiag (Ospedale San Camillo, Roma), Francesca Izzi (AOU Tor Vergata, Roma), Angela La Neve (Ospedale Consorziale Policlinico, Bari), Emilio Le Piane (Ospedale Pugliese, Catanzaro), Concetta Luisi (Policlinico Ospedaliero, Padova), Greta Marcorig (Ospedale Civile, Gorizia), Carlo Alberto Mariani (ASP, Palermo), Carla Marini (Ospedale Salesi, Ancona), Alfonso Marrelli (Osp. Reg, S, Salvatore, L’Aquila), Marta Maschio (**Center for Tumor Epilepsy, UOSD Neuroncologia IRCCS Regina Elena, Rome**), Roberto Michelucci (Ospedale Bellaria, Bologna), Fabio Minicucci (IRCCS San Raffaele, Milano), Antonio Modica (ARNAS Civico Di Cristina, Palermo), Maurizio Montalto (AO Riuniti Villa Sofia Cervello, Palermo), Francesca Muzzi (Policlinico Grassi, Ostia, Roma), Rosaria Nardello (Policlinico, Univ. Giaccone, Palermo), Alessandro Orsini (Ospedale Santa Chiara, Pisa), Nicola Paciello (Ospedale San Carlo, Potenza), Mariangela Panebianco (Opsedale Garibaldi, Catania), Irene Pappalardo (Ospedale San Martino, Genova), Daniela **Passarelli** (Ospedale, Faenza, Ravenna), Giada Pauletto (Ospedale S.M. Misericordia, Udine), Piero Penza (AOU Ruggi D’Aragona, Salerno), Gabriella Perri (Ospedale Salvini **Rhodense**, Garbagnate, Milano), Marianna Pezzella (Ospedale Cardarelli, Napoli), Piero Pignatta (Ospedale Gradenigo, Torino), Dario Pruna (AO Brotzu, Cagliari), Stefano Quadri (Ospedale Giovanni XXIII, Bergamo), Rosaria Renna (AORN Cardarelli, Napoli), Sara Renzi (Ospedale Madonna del Soccorso, Ascoli), Paolo Ricciardelli (Ospedale, Faenza, Ravenna), Romana Rizzi (AO Santa Maria Nuova, Reggio Emilia), Angelo Russo (Ospedale Bellaria NPI, Bologna), Nicola Sciscio (Centro Australia, Avellino), Vittorio **Sciruicchio** (Policlinico San Paolo, Bari), Carlotta Spagnoli (AO Santa Caterina Nuova, Reggio Emilia), Orazio Spitaleri (Pres. Osp. *S. Marta* S. Venera, Acireale, Catania), Pasquale Striano (Istituto Gaslini, Genova), Elena Tartara (Istituto Neurologico Mondino, Pavia), Lidia Urso (AO Sant’Antonio Abate, Erice, Trapani), Anna Vaudano (Ospedale, Baggiovara, Modena), **Pierangelo** Veggiotti (Ospedale Buzzi, Milano), Fabiana Vercellino (Ospedale San Biagio, Alessandria), Maurizio Viri (Ospedale Maggiore, Novara), Roberta Vittorini (OIRM Regina Margherita, Torino), Cristina Zammarchi (Ospedale Infermi, Rimini), Clara Zanchi (Ospedale, Seriate, Bergamo), Tiziano Zanoni (Osp. Borgo Trento, Verona), **Lucia** Zinno (Ospedale Maggiore, Parma), Leila Zummo (ARNAS Civico Di Caterina, Palermo), **Nicoletta Foschi (Ospedale Torrette, Ancona)**.

The original article [1] has been updated.

## References

[1] Bonanni P, Gambardella A, Tinuper P (2021). Perampanel as first add-on antiseizure medication: Italian consensus clinical practice statements. BMC Neurol.

