# Supplementary material for: Perampanel as first add-on antiseizure medication: Italian consensus clinical practice statements
Source: BMC Neurol. 2021 Oct 26;21:410. doi: 10.1186/s12883-021-02450-y (PMC8549193; doi:10.1186/s12883-021-02450-y)
Supplement: Supplementary file 1 — Additional file 1: Supplementary Table 1. General statements and statements addressing efficacy and safety issues, with associated ratings for relevance and level of agreement. Supplementary Table 2. List of statements addressing potential implications of drug interactions, mechanism of action and adherence, with associated ratings for relevance and level of agreement. Supplementary Table 3. List of statements addressing potential implications of ease of use and other factors specific for the Italian setting, with associated ratings for relevance and level of agreement. [file 12883_2021_2450_MOESM1_ESM.docx]

Supplementary Table 1. General statements and statements addressing efficacy and safety issues, with associated ratings for relevance and level of agreement.

| **Statements** | **Consensus**  **(% of ratings in the 7-9 bracket)** | **Relevance**  **rating**  **(1-3)** |
| --- | --- | --- |
| ***General statement*** | | |
| Perampanel is indicated for use as add-on therapy | Yes (97.6%) | 2.8 |
| With respect to favorable retention data shown in clinical studies, including long term studies, perampanel has been shown to ensure a good quality of life due to its efficacy, tolerability and ease of use | Yes (94.0%) | 2.8 |
| ***Statements related to efficacy*** | | |
| Perampanel is effective against focal and generalized tonic-clonic seizures | Yes (91.7%) | 2.9 |
| Perampanel has not been shown to worsen other types of seizures such as myoclonus or absences | Yes (91.7%) | 2.8 |
| Perampanel may be useful when there is uncertainty as to whether seizures are focal or generalized | Yes (91.7%) | 2.9 |
| Recent observational studies have shown that perampanel used as add-on therapy can be effective and well tolerated even at low doses, such as 4 and 6 mg /day | Yes (95.2%) | 2.9 |
| Perampanel can be effective and well tolerated when used either early or late as add-on therapy | Yes (81.0%) | 2.7 |
| ***Statements related to tolerability and safety*** | | |
| Short- and long-term tolerability and safety have been found to be good. In the long term, no iatrogenic (developmental and cognitive) or adverse effects have emerged, other than those observed in the short term | Yes (90.4%) | 2.9 |
| Perampanel, like all drugs, can cause dose-dependent adverse effects. Use of low doses and a slow rate of titration improve tolerability | Yes (96.4%) | 2.9 |
| The most frequent adverse effects of perampanel are dizziness, somnolence, headache, fatigue, irritability. Tolerability is better when perampanel is associated with fewer ASMs | Yes (96.4%) | 2.8 |
| With respect to perampanel's behavioral and psychiatric tolerability profile, the most common adverse effect is irritability. | Yes (94.0%) | 2.7 |
| As for all ASMs, the adverse effects of perampanel, especially those affecting the central nervous system, occur more commonly in the first weeks and tend to decrease (tolerance) with the continuation of therapy | Yes (92.9%) | 2.6 |
| As with other ASMs, when using perampanel it is important to consider the patient's comorbidities at baseline. | Yes (95.2%) | 2.8 |
| With respect to perampanel’s behavioral and psychiatric tolerability profile, it is advisable to carefully evaluate and monitor its use in patients with these problems at baseline or in their medical history. | Yes (94.0%) | 2.8 |
| Available scientific evidence has not demonstrated significant adverse effects of perampanel on cognitive functions in the short and medium term in either adolescents or adults | Yes (94.0%) | 2.9 |
| Although sleepiness is an adverse effect associated with the use of perampanel, the available scientific evidence based on studies that assessed sleep through specific tests has shown that perampanel does not worsen daytime sleepiness and the quality of night sleep in most patients | Yes (93.8%) | 2.7 |
| Perampanel is metabolised in the liver and is subject to enzyme induction | Yes (80.8%) | 2.6 |
| Perampanel does not affect adversely cardiac electrophysiology | Yes (96.4%) | 2.9 |
| There are currently no data on the safety of perampanel in pregnancy, particularly with regard to seizure control and possible effects on the offspring. | Yes (96.4%) | 2.9 |

Supplementary Table 2. List of statements addressing potential implications of drug interactions, mechanism of action and adherence, with associated ratings for relevance and level of agreement.

| **Statement** | **Consensus**  **(% of ratings in the 7-9 bracket)** | **Relevance**  **rating**  **(1-3)** |
| --- | --- | --- |
| ***Statements related to drug interactions*** | | |
| When adding perampanel to a pre-existing monotherapy, it is worth considering whether or not an inducer is present because a higher dose of perampanel may be needed | Yes (80.7%) | 2.6 |
| When using perampanel as add-on therapy, it may be possible to reduce the dosage of concomitant ASMs, in order to optimize tolerability. | Yes (89.3%) | 2.6 |
| Perampanel has a limited number of drug interactions, generally of modest clinical significance | Yes (94.0%) | 2.8 |
| An increased risk of adverse effects when perampanel is combined with specific ASMs or ASM classes has not been documented in the clinical setting. | Yes (94.0%) | 2.6 |
| To date, no evidence has emerged of drug interactions that could lead to a worsening of the tolerability of perampanel. | Yes (93.8%) | 2.6 |
| ***Statements related to mechanisms of action*** | | |
| Perampanel has a unique mechanism of action which is complementary to that of other ASMs currently on the market. | Yes (97.6%) | 2.9 |
| ***Statements related to adherence issues*** | | |
| Perampanel has a long plasma half-life which allows once daily administration | Yes (98.8%) | 2.9 |
| Perampanel has a simple dosing schedule: one tablet once daily for each dosing level | Yes (98.8%) | 2.9 |
| The possibility of taking perampanel in the evening at bedtime is convenient for the patient | Yes (97.6%) | 2.8 |
| Regular compilation of the therapeutic plan for perampanel can represent an indicator of drug intake and, therefore, correct therapeutic adherence | No (61.5%) | 2.3 |

Supplementary Table 3. List of statements addressing potential implications of ease of use and other factors specific for the Italian setting, with associated ratings for relevance and level of agreement.

| **Statement** | **Consensus**  **(% of ratings in the 7-9 bracket)** | **Relevance**  **rating**  **(1-3)** |
| --- | --- | --- |
| ***Statements related to ease of use and related factors*** | | |
| Perampanel is advantageous because of its once daily administration | Yes (97.6%) | 2.9 |
| Perampanel does not require multiple administration: each dosage level is covered by one specific tablet to be taken | Yes (97.6%) | 2.8 |
| The efficacy characteristics of perampanel (and an adequate titration) allow in many cases its use at low maintenance doses, starting from 4 mg/day | Yes (90.5%) | 2.9 |
| Perampanel does not require routine repeated blood chemistry or laboratory investigations in the majority of patients | Yes (96.4%) | 2.7 |
| Perampanel is available in different formulations, i.e. as tablets and oral suspension | Yes (95.2%) | 2.7 |
| Should therapeutic drug monitoring be desirable, measurements of plasma perampanel levels are not widely accessible | Yes (80.8%) | 2.3 |
| ***Peculiarities to the Italian setting*** | | |
| Distribution through the DPC channel (‘*distribuzione per conto’*, i.e. acquisition and distribution to pharmacies handled by local health authorities) guarantees the availability of perampanel and avoids problems with shortage of medication | YES (88.0%) | 2.6 |
| The different packages of perampanel tablets have the same price, regardless of the dose level, which guarantees the same cost to the national health service regardless of the dosage taken by the patient. | YES (94.8%) | 2.6 |
| The DPC distribution of drugs that are included in the PHT (‘*prontuario ospedale territorio’)* formulary, such as perampanel, guarantees greater cost-effectiveness for the health service compared to non-PHT drugs. which mainly use the traditional distribution channel | YES (81.9%) | 2.5 |
| Compilation of the therapeutic plan, while representing an inconvenience, provides a tool that helps to ensure appropriateness in perampanel prescribing | NO  (69.2%) | 2.3 |
